# Association between Chronological Age and IGF-1, IGFBP-3, and CTX Levels in Saliva of Children through Younger Adult Population with Varying Periodontal Status

**DOI:** 10.3390/children9091301

**Published:** 2022-08-27

**Authors:** Abdullah Almalki, Julie Toby Thomas, Saud Alotaibi, Mansour Alasiri, Hamdan Alamri, Mohamed Helmy Salama

**Affiliations:** 1 Department of Preventive Dental Sciences, College of Dentistry, Majmaah University, Al-Majmaah 11952, Saudi Arabia; 2 Department of Periodontics, College of Dentistry, Al-Azhar University, Cairo 11651, Egypt

**Keywords:** bone remodeling, insulin-like growth factor-1, IGF-binding protein-3, cross-linked C-terminal telopeptide of type I collagen, periodontitis

## Abstract

The quest for the most precise and non-invasive technology to monitor the pubertal growth spurt is driven by the role of growth determination in orthodontics. The objective of this study was to estimate the levels of salivary insulin-like growth factor-1 (IGF-1), IGF-binding protein-3 (IGFBP-3), and cross-linked C-terminal telopeptide of type I collagen (CTX1), and to analyze whether the levels of these biomarkers vary among different chronological age groups with and without periodontal disease. Eighty participants were divided into three groups based on their chronological age: group 1: 6–12 years; group 2: 13–19 years; and group 3: 20–30 years. The assessed clinical parameters included the simplified oral hygiene index (OHI-S), bleeding on probing (BOP), probing pocket depth (PPD), clinical attachment loss (CAL), and community periodontal index (CPI). Using ELISA kits, the IGF-1, IGFBP-3, and CTX1 levels in the saliva samples were estimated. The salivary concentration of IGFBP-3 was significantly associated with age and gender (*p* < 0.01). However, no significance was observed between subjects with and without periodontal disease. Significant associations existed between the values of IGF-1, IGFBP-3, and CTX1 in saliva among subjects from the various chronological age groups. Estimation of salivary IGF-1 and IGFBP-3 could serve as a useful tool in the assessment of growth maturity and bone remodeling patterns during orthodontic treatment planning.

## 1. Introduction

A proper evaluation of the maturational stage during diagnosis and treatment is essential for successful orthodontic therapy. An integrative collaboration between orthodontic and periodontal care promotes the attainment of desired outcomes in the clinical management of malocclusion. Healthy and sound periodontal tissues minimize the detrimental implications of excessive bone resorption, which is clinically manifested as gingival recession, bony defects such as dehiscence, fenestrations, and loss of clinical attachment during the phase of tooth activation. The relevance of periodontal examination before, during, and after active orthodontic therapy is highly warranted when dealing with compromised cases concerning pathologic tooth migration, intra-bony defects, and occlusion-related damage [1].

Human saliva helps with a variety of other body functions, such as tissue maintenance, oral protection, swallowing, food taste, and digestion, but in the last ten years, the use of saliva as a diagnostic tool has grown significantly. Clinicians and researchers employ human saliva because of its ease of transportability, simple collection techniques, ease of disposal, cost effectiveness, and increased patient compliance [2]. Salivary diagnostics requires appropriate identification and validation of biomarkers for the detection of diseases. Including serum, saliva also has hormones, antibodies, growth factors, enzymes, microorganisms and their by-products, genetic material (such as DNA and RNA), and protein molecules that reflect the physiological status of an individual [3].

Periodontal disease is a public health concern due to its high prevalence in adolescents, adults, and the elderly. Clinical cases of malocclusion are common (88%) and have been considered as one of the predisposing factors for periodontal disease. Past history of periodontal disease, poor dental hygiene, and smoking are all variables that make patients more vulnerable to periodontitis [4]. Active periodontal disease caused by tooth biofilm can hasten periodontal deterioration by influencing the bone remodeling process. As a result, monitoring of periodontal indicators in addition to skeletal maturity assessment as part of a routine orthodontic diagnostic approach is essential to achieving optimal orthodontic clinical outcomes.

Investigators have elucidated various growth assessment methods to establish the appropriate timing of tooth movement such as body height, weight, sexual maturation, frontal sinus, chronological age, physiological age, hand-wrist maturity, cervical vertebrae, dental eruption, dental calcification stages, and growth biomarkers [5,6,7,8,9,10]. Although chronological age can be used to predict adulthood, it is a crude indicator of biological maturation processes that do not necessarily correspond to a child’s developmental stage. Despite the fact that bone age assessment is still a standard radiological procedure for pediatrics, it takes time and requires radiologist expertise, with intra- and interobserver variability resulting in bone age and chronological age discrepancies [11].

Molecular biology has enabled researchers to shed light on the function of biological growth mediators such as insulin-like growth factors (IGF-1), IGFBP-3, alkaline phosphatase (AP), and osteocalcin (OC) in estimating pubertal maturity. Through the use of these biomarkers in the future, orthodontic treatment may be avoided, incurring undesired additional radiation exposures. Bone remodeling is influenced by the upregulation of circulating growth hormone (GH), IGFs, IGFBPs, and locally produced IGFs and IGFBPs that act on bone matrix receptors to drive osteoblast differentiation [12,13]. IGFs are present in blood serum, gingival crevicular fluid (GCF), or saliva, and are typically bound to IGFBP-1 to -6. IGFBP-3 is the predominant IGFBP that regulates the quantity of free, bioactive IGF-1, therefore playing a role in bone cell proliferation. According to research by Kanbur et al., the peak of the serum IGF-1/IGFBP-3 molar ratio corresponds to the time when bone synthesis is accelerated during the pubertal growth spurt. As a result, they came to the conclusion that the IGF-1/IGFBP-3 molar ratio might be used to predict growth spurts accurately [14].

There is evidence that the severity of periodontal disease is related with decreased levels of IGFBP-3 in the blood [15]. The presence of IGF-I and its receptors in periodontal tissues modulates the immune response by inducing B cell growth, immunoglobulin synthesis, and interleukin-6 (IL-6) production during inflammation. Disruption of the homeostasis of osteoclastic and osteoblastic activity is linked to alveolar bone loss. Various biomarkers of bone turnover include alkaline phosphatase (AP), osteocalcin (OC), C-terminal propeptide of type I procollagen (PICP), cross-linked C-terminal of type I collagen (ICTP), cross-linked C-terminal telopeptide of type I collagen (fragments alpha-CTX, beta-CTX), and N-terminal propeptide of type I procollagen (PINP) [16].

During bone resorption, the insoluble collagen type 1 collagen fragments in the resorption compartment of the osteoclast and the C-terminal telopeptide region of type I collagen (CTX) are expressed in the body fluids. CTX levels in oral fluids have been shown to be a predictable diagnostic marker in assessing periodontal disease activity, with good sensitivity and specificity for detecting increasing bone damage [17]. According to a study conducted by Joseph et al. in 2019, salivary CTX, OC, and ON (osteonectin) concentrations can distinguish healthy participants from those with periodontitis [18]. The investigation demonstrated by Choi YJ et al. 2020 analyzed the role of insulin-like growth factor 1 and insulin-like growth factor-binding protein-3 in identifying growth hormone deficiency. They found that both IGF-1 and IGFBP-3 correlated well with chronological age compared to bone age and pubertal age, suggesting its future implications as a screening tool [19].

In view of the present literature, it is important to emphasize the importance of periodontal diagnosis in orthodontic treatment planning and the feasibility of using non-invasive salivary biomarkers to predict periodontal tissue damage during orthodontic therapy. Therefore, the present study aimed to evaluate salivary IGF-1, IGFBP-3, and CTX among children, adolescents, and younger adults and to determine the association of these markers among the study population with different chronological age groups with and without periodontal disease.

## 2. Materials and Methods

This cross-sectional study was conducted at Majmaah University, Zulfi, Saudi Arabia, between December 2021 and February 2022 after being approved by the institutional ethical committee of Majmaah University, Saudi Arabia (Research Number: MUREC Nov.08/COM-2020/8-2; dated 8 November 2020) in accordance with the Helsinki Declaration.

In total, 110 patients, aged 6–30, who sought orthodontic consultation at the outpatient department of preventive dentistry, were randomly selected for this study. We excluded patients with chronic systemic diseases; diseases affecting growth such as vitamin D deficiency; parathyroid, growth, and thyroid hormone disorders; renal disorder; diabetes; growth abnormalities; blood disorders; those taking bone metabolism medications for the past six months; xerostomia; those who reported previous orthodontic treatment; radiotherapy; pregnant or lactating patients; and smokers. Before being enrolled, each participant over the age of 14 and the caregivers of participants under the age of 14 signed an informed consent form. Sixteen subjects refused to cooperate during saliva collection, and fourteen subjects did not approve of written consent, resulting in a total of thirty dropouts. All data was recorded and statistically analyzed for 80 subjects.

A personal interview was conducted for all the participants enrolled. Sociodemographic data comprising age, gender, nativity, previous medical and dental history, a family history of periodontal disease, and personal oral hygiene habits were electronically recorded by a single researcher. The subjects were classified into three groups: group 1: 6–12 years old; group 2: 13–19 years old; and group 3: 20–30 years old.

A mouth mirror and a community periodontal index probe (CPI) were used to examine and record periodontal variables. An intra-examiner calibration value of 0.86 was estimated using kappa statistics on 15 study subjects assessed by the initial examiner. To evaluate the oral health status of all study subjects, the simplified oral hygiene index (OHI-S), bleeding on probing (BOP), probing pocket depth (PPD), clinical attachment loss (CAL), and community periodontal index (CPI) were estimated [20,21,22]. Apart from BOP, the periodontal parameters such as PD and CAL assessed were strictly confined to fully erupted permanent dentition.

As per the World Health Organization (WHO) 1997 guidelines, the whole dentition was divided into six sextants. From each sextant, one index tooth was chosen (16, 11, 26, 36, 31, and 46). The scores assigned to the subjects were based on the following criteria: CPI = 0, normal; CPI = 1, bleeding on probing and no pocket 3.5 mm; CPI = 2, calculus present and no pocket 3.5 mm; CPI = 3, shallow pocket 3.5–5.5 mm; and CPI = 4, deep pocket 5.5 mm. The highest score was taken into consideration for the assessment of subjects’ periodontal status [21].

For the assessment of OHI-S, the lingual and buccal surfaces of teeth 16, 26, 11, and 31, and the lingual surfaces of teeth 36 and 46 were inspected. The index tooth evaluated for evaluating the simplified oral hygiene index in the case of deciduous dentition included the labial surface of 54, 61, and 82, and the lingual surface of 75. In the case of mixed dentition, the labial surface of 26 and the lingual surface of 46 were also included. Each subject’s OHI-S score was calculated by adding the debris index and calculus index values. The debris index and calculus index were calculated by summing the individual values and dividing them by the total number of teeth evaluated after recording individual tooth scores for debris and calculus. The outcomes were divided into three categories: excellent (0–1.2), fair (1.3–3), and poor (3–16) [20].

The PD measurement was recorded from the gingival margin to the base of the sulcus by probing all six sites of the tooth (mesiobuccal, mid-buccal, distobuccal, mesiolingual, mid-lingual, and distolingual). During the process of recording, bleeding at any site was also noted 10–15 s after probing. The presence of BOP in the specific site was indicated as a positive sign (+) in the patient data. Patients with BOP in more than 10% of the sites were categorized as having gingivitis. The distance between the cementoenamel junction and the base of the gingival sulcus was used to calculate the CAL. Participants with CAL were considered to meet the criteria for periodontitis.

All participants were instructed to report between the hours of 10 a.m. and 12 a.m. to standardize the diurnal variation. Before accumulating saliva in the mouth, they were told to sit straight in a comfortable position and completely rinse their mouths. In total, 5 mL of unstimulated saliva was collected in a graded Eppendorf tube using the spitting method [23]. All of the tubes were labeled by the primary investigator, and the samples were stored at −20 °C until further analysis.

Within four months, collected samples were tested for IGF-1 and IGFBP-3 using an enzyme-linked immunosorbent assay (ELISA). To eliminate suspended particles, frozen saliva was thawed and centrifuged for 10 min at 3500 RPM; the clear supernatant was used to estimate IGF-1 and IGFBP-3. Commercially available ELISA kits for IGF-1 (Product No. SEA050Hu) and IGFBP-3 (Product No. SEA054Hu) were obtained from Cloud-Clone Corp, Katy, TX, USA and were utilized according to the manufacturer’s instructions.

The saliva sample solution and 200 μL (microliters) of the standard solution were pipetted into each well of the pre-coated ELISA plate and incubated at 37 °C for 1 h. Each well had its clear liquid removed. Then, 200 μL of the kit’s “prepared detection reagent A” was added. This was combined, incubated for 1 h at 37 °C, aspirated, and washed 3 times. After this, 200 μL of “prepared detection reagent B” was added, incubated for 30 min at 37 °C, then aspirated, and cleaned 5 times. In total, 180 μL of substrate solution was added to this and incubated for 10–20 min at 37 °C. Thereafter, 100 μL of stop solution was added, and the optical density (OD) was measured at 450 nm with an ELISA reader [24].

The participants’ data was summarized using appropriate statistical techniques. The study’s continuous variables, such as age and biochemical parameters, were summarized using the mean and standard deviation. The frequency and percentage were chosen as summary measurements for categorical variables. We compared the biochemical parameters between groups using an independent t-test and one-way ANOVA with Tukey post hoc tests. We also used the Pearson and Spearman correlation to evaluate the correlation between the age and clinical parameters with the biochemical parameters. Multiple linear regression was used to assess the relationship between the biochemical parameters and variables (age, sex, and periodontitis). SPSS version 28 (IBM Corp. Armonk, NY, United States) was used for all analyses, and the statistical significance was set at the 5% level.

## 3. Results

Out of 110 subjects, aged 6 to 30, data from 80 included participants was considered for the analysis. Table 1 describes the demographic characteristics of the participants. The majority of the participants were older than 20 years (*n* = 39) and belonged to the female gender (*n* = 50) from rural areas. However, participants older than 20 years had a significantly higher proportion of periodontitis compared to other age groups.

More participants from rural backgrounds had gingivitis and periodontitis (63.2% and 66.7%) compared to urban (36.8% and 33.3%), but the differences in the results were not found to be statistically significant. The proportions of female participants diagnosed with gingivitis (52.6%) and periodontitis (58.3%) were higher than the percentages of male participants (47.4% and 41.7%), but the differences were not statistically significant.

We compared the biochemical parameters IGF-1, IGFBP-3, IGF-1/IGFBP-3 molar ratio, and collagen telepeptidase-1 (CTX1) between the age groups using one-way ANOVA with Tukey post hoc tests (Table 2). Levels of IGF-1, IGF-1/IGFBP-3 ratio, and CTX1 showed statistically significant differences between the different chronological age groups. In comparison to the 6–12 age group, the levels of IGF-1, IGFBP-3, and IGF-1/IGFBP-3 molar ratio in the saliva of 13–19-year-olds were significantly higher. Levels of CTX1 were significantly higher among ≥20 years group.

**Figure 1:**
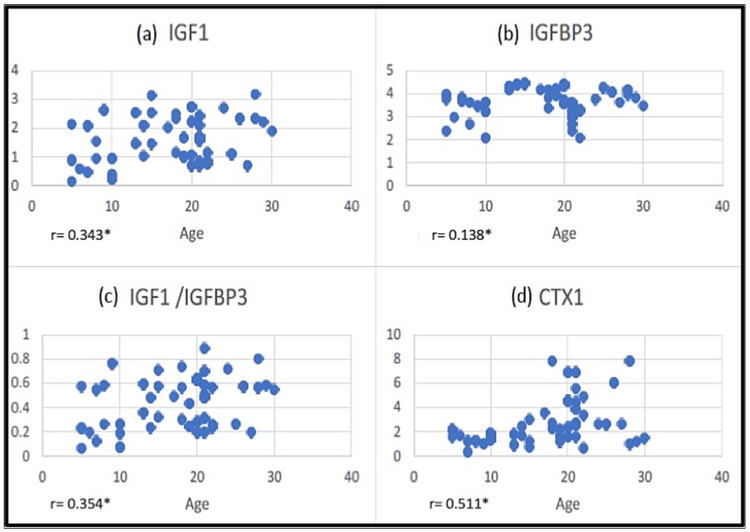
Correlation between ages with (**a**) IGF-1, (**b**) IGFBP-3, (**c**) IGF-1/IGFBP-3, and (**d**) CTX1. * Statistically significant.

There was a significant positive correlation between the levels of IGF-1 and IGFBP-3 and IGF-1 and IGF-1/IGFBP-3 molar ratio, with an R-value of 0.453 and 0.939, respectively. A significant positive correlation was found between various chronological age groups and salivary IGF-1, IGF-1/IGFBP-3 molar ratio, and CTX-1, except IGFBP-3 (Figure 1).

Table 3 shows a comparison of the salivary levels of the parameters analyzed between males and females. None of the biochemical parameters were different between the sexes except IGFBP-3, which was significantly higher among males.

**Figure 2:**
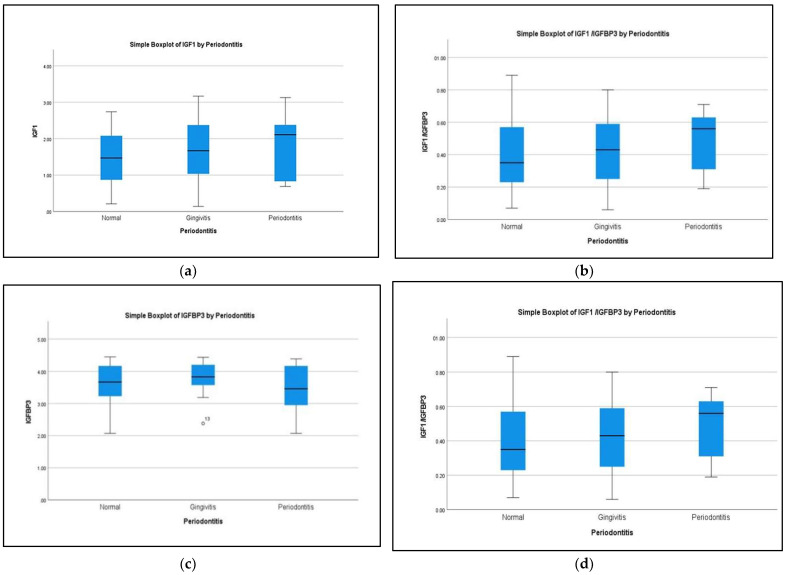
Comparison of the biochemical parameters between healthy participants and those with periodontal disease: (**a**) IGF-1, (**b**) IGFBP-3, (**c**) IGF-1/IGFBP-3, and (**d**) CTX1.

In Figure 2, biochemical parameters are compared between subjects with healthy periodontium, gingivitis, and periodontitis. There were no differences in any of the biochemical parameters between these groups. The levels of CTX were higher among participants with periodontitis. However, the difference was not statistically significant (*p* = 0.075).

No significant correlation was found between the clinical parameters, OHI-S, BOP, PPD, CAL, CPI, and salivary parameters estimated (Table 4).

Multiple linear regression analysis with the biochemical parameters as the dependent variable and age, gender, and periodontal condition as the independent variables showed that age was a significant predictor of IGF-1, IGF-1/IGFBP-3, and CTX-1 (*p* < 0.001). Gender was a significant predictor of IGFBP-3 (*p* = 0.006). However, none of the biochemical parameters were significantly related to periodontal disease severity (Table 5).

## 4. Discussion

The health of periodontal tissues can influence the success of orthodontic treatment. A periodontal marker that can assess periodontal health will be very helpful for the enhancement of clinical examinations for periodontal diseases that often go unnoticed. It is particularly important when planning orthodontic treatment for aged patients who may be suffering from early stage periodontal disease. The malalignment of teeth can influence the severity of periodontal disease, thus emphasizing the importance of a multidisciplinary approach. Furthermore, studies have shown that patients with severe malocclusion have lower oral health-related quality of life scores than those with less critical treatment needs [4]. It is therefore imperative to assess gingival and oral hygiene and the health of the attached gingiva in orthodontic patients. The present study evaluated the markers of IGF-1, IGFBP-3, and CTX1 among young adults seeking orthodontic treatment.

For an orthodontist, the adolescent growth spurt coincides with periods of rapid growth because the best time to modify facial growth is at the onset of the growth spurt when the levels of facial growth are at their maximum [25]. In response to growth hormone, IGF-1 (Somatomedin C) is produced in the liver, which is crucial for bone formation. To obtain the best results in orthodontics and orthognathics, the estimation of IGF-1 could also identify growth abnormalities and anticipate skeletal development through puberty. The comparison of the salivary levels of IGF-1 and IGFBP-3 between different age groups in this study demonstrated significantly higher levels among the 13–19-year age group, compared to both the 6–12-year and ≥20-year age groups. These results are in accordance with previous studies. Almalki et al. (2022) demonstrated that quantitative estimation of IGF-1 and IGFBP-3 in saliva and their molar ratio can play an essential role in the assessment of bone maturity. They found that from prepuberty to the onset of puberty, salivary IGF-1 levels increased gradually before a sharp decline at the pubertal peak [13].

The dominant binding protein for IGF-1 in the blood is IGFBP-3. Despite its protective function of blocking IGF-1’s mitogenic effect on cell growth, IGFBP-3 provides a precise estimate of the amounts of free IGF-1 present in biological fluids. By directly interacting with extracellular and cell surface molecules on mineralized tissues, IGFBPs also perform independently of IGF-1, establishing their involvement in bone modeling. The significant increase in salivary IGFBP-3 between the ages of 13 and 19 years in this study is consistent with the findings reported by Juul et al. in 1995, where they reported a peak in serum IGFBP-3 levels during pubertal age [26].

Considering the fluctuation of IGF-1 and IGFBP-3 levels in saliva with chronological age, gender, height, and body mass index, calculation of the molar ratio of IGF-1 to IGFBP-3 would provide a good indication of the increase in free biologically active IGF-1. The increase in the salivary IGF-1/IGFBP-3 molar ratio between the ages of 13 to 19 years in this study is consistent with the results reported by Kanbur NÖ et al., where a maximal increase in the serum IGF-1/IGFBP-3 molar ratio influenced the onset of the pubertal growth spurt, which is congruent with the accelerated rate of skeletal development during puberty [14].

Except for IGFBP-3, there was a significant positive correlation between salivary IGF-1, IGF-1/IGFBP-3 molar ratio, and CTX-1 in various chronological age groups. No gender differences in the salivary IGF-1, IGF-1/IGFBP-3 molar ratio, or CTX-1 were observed in this study except for the levels of IGFBP-3, which were significantly higher among males. The results of this study are consistent with the study carried out by Lofqvist C et al., who found that blood IGFBP-3 levels increased with age in boys but remained constant in girls during mid-pubertal growth. Evidence-based research demonstrates that different nutritional variables and IGF-1 bioavailability are altered by IGFBP-3 promoter polymorphisms, resulting in a variable concentration of IGFBP-3 [27].

The severity of periodontal tissue destruction depends on the chronicity of the periodontal disease, which was found to be rare among the younger age group, which could be the possible reason for significantly higher levels of CTX1 among the chronologic age group of more than 20 years. The demographic and clinical data estimated in this study represent more subjects affected by periodontitis in this age group compared to the other age groups. The findings of this study are in accordance with the study reported by Betsy et al., where the CTX concentrations in saliva were higher in subjects with periodontitis compared to healthy subjects [18]. The positive correlations of salivary CTX with age observed in the present study corroborate the findings reported by Mishra et al. [16]. Among all the biochemical parameters, only the CTX level was higher among participants with periodontitis. As a marker of bone turnover, serum C-telopeptide cross-link of type 1 collagen (sCTX) is a highly sensitive indicator of increased bone resorption. Investigators have reported linking elevated CTX levels with increased periodontal disease severity [28,29].

Inflammation has a negative effect on the growth marker-binding protein IGFBP. However, in this study, the correlation between the clinical parameters assessed using OHIs, BOP, PPD, CAL, and CPI with biochemical parameters did not demonstrate any significant association. These findings were contradictory to the findings reported by Takenouchi et al., where they found a positive correlation between the concentration of IGFBP-2 and the probing depth and gingival index but not for IGFBP-3 [30]. It has been shown that IGFBP-3 regulates epidermal homeostasis by localizing to the nucleus of numerous cultivated cell lines, including epidermal keratinocytes, perhaps by influencing the early stages of keratinocyte terminal differentiation. These findings indicate that IGFBP-3 is linked to periodontal tissue homeostasis rather than being engaged in periodontitis progression. Since the study participants in this study belonged to younger age groups, it is considerably rare for them to have severe generalized periodontitis. The possible reason for the significant rise in the levels of IGFBP-3 could be its influence on tissue homeostasis. However, further studies are warranted to substantiate the reported findings. Another contributing factor that could affect the findings reported in this study might be the significantly higher proportion of periodontitis participants aged more than 20 years compared to other age groups.

Age was a significant predictor of IGF-1, IGF-1/IGFBP-3, and CTX-1 in a multiple linear regression analysis using biochemical parameters as the dependent variable and age, gender, and periodontal condition as the independent variables. Gender was found to be a significant predictor of IGFBP-3 levels. A comparison of the biochemical parameters IGF-1, IGF-1/IGFBP-3, and CTX1 found no statistically significant difference between sexes. Though surprising, it is possible as the sample had almost twice the number of females compared to males in all the age groups, whose growth period is earlier than males of the same chronological age.

In the multiple regression analysis, age to a greater extent and gender to some extent influenced the levels of biochemical parameters but periodontal conditions did not. In addition to bivariate analysis, this further confirms that in growing phases, the levels are unreliable. Thus, interpretation of these marker levels should be made with caution. However, the drawback of this study is the lack of sufficient samples pertaining to severe generalised periodontitis and the decreased expression of these markers in saliva, which could have affected the findings of this study. Further longitudinal research needs to be conducted with a large group of individuals with varying severity of periodontitis for better internal validity.

This study was conducted among randomly selected healthy orthodontic patients aged 6–30 years with no systemic disorders seeking treatment in an outpatient clinic. Hence, despite limitations, the results of this study are confined to the Saudi population under the age of 30 years and a larger sample population is required to generalize the study’s validity in the future.

## 5. Conclusions

The salivary levels of IGF-1, IGFBP-3, and IGF-1/IGFBP-3 molar ratio in 13–19-year-olds were significantly higher compared to the other age groups. Estimation of salivary IGF-1 and IGFBP-3 could serve as a beneficial tool in determining growth maturity and bone remodeling during orthodontic treatment planning. There were no differences in the salivary biochemical parameters between subjects with healthy periodontium, gingivitis, and periodontitis. The levels of salivary CTX1 were significantly higher among the ≥20-years group. The levels of CTX were higher among participants with periodontitis. Salivary CTX levels in periodontitis patients are influenced by age and can be used as a reliable predictor to monitor periodontal disease severity in patients undergoing orthodontic treatment.

## Figures and Tables

**Table 1:** Demographic characteristics of the participants.

Variable Class		Periodontal Disease			Total (*n*)%	*p* Value (Chi-Square Test)
		Normal (*n*)%	Gingivitis (*n*)%	Periodontitis (*n*)%		
**Age**	6–12 years	20	2	0	22	0.006 *
		40.8%	10.5%	0.0%	27.5%	
	13–19 years	10	7	2	19	
		20.4%	36.8%	16.7%	23.8%	
	≥20 years	19	10	10	39	
		38.8%	52.6%	83.3%	48.8%	
**Gender**	Male	16	9	5	30	0.504
		32.7%	47.4%	41.7%	37.5%	
	Female	33	10	7	50	
		67.3%	52.6%	58.3%	62.5%	
**Location**	Urban	14	7	4	25	0.793
		28.6%	36.8%	33.3%	31.3%	
	Rural	35	12	8	55	
		71.4%	63.2%	66.7%	68.8%	

* Statistically significant.

**Table 2:** Comparison of biochemical parameters between different age groups.

Parameter	Age (Years)	*n*	Mean	Std. Deviation	Std. Error	95% Confidence Interval for Mean		*p* Value (One Way ANOVA)
						Lower Bound	Upper Bound	
**IGF-1**	6–12 ^†^^¥^	22	0.99	0.82	0.17	0.63	1.35	<0.001 *
	13–19 ^¥^	19	1.82	0.65	0.15	1.51	2.14	
	≥20 ^†^	39	1.73	0.76	0.12	1.49	1.98	
**IGFBP-3**	6–12 ^†^	22	3.32	0.56	0.12	3.07	3.57	<0.001 *
	13–19 ^†^^¥^	19	4.17	0.27	0.06	4.04	4.30	
	≥20 ^¥^	39	3.56	0.57	0.09	3.38	3.75	
**IGF-1/IGFBP-3**	6–12 ^†^^¥^	22	0.29	0.23	0.05	0.19	0.39	0.001 *
	13–19 ^¥^	19	0.44	0.16	0.04	0.36	0.52	
	≥20 ^†^	39	0.49	0.19	0.03	0.42	0.55	
**CTX1**	6–12 ^†^	22	1.41	0.51	0.11	1.19	1.64	<0.001 *
	13–19 ^¥^	19	2.14	1.57	0.36	1.38	2.89	
	≥20 ^†^^¥^	39	3.85	2.03	0.33	3.19	4.51	

The same superscript in front of 2 age groups († or ¥) in column 2 indicates the difference is statistically significant between respective groups as found in the post-\ hoc test (Dunnette) * Statistically significant.

**Table 3:** Comparison of the biochemical parameters among genders.

Parameters	Gender	N	Mean	Std. Deviation	Std. Error Mean	*p* Value (Independent *t* Test)
**IGF-1**	Male	30	1.76	0.89	0.16	0.069
	Female	50	1.42	0.75	0.11	
**IGFBP-3**	Male	30	3.92	0.32	0.06	<0.001 *
	Female	50	3.47	0.66	0.09	
**IGF-1/IGFBP-3**	Male	30	0.44	0.22	0.04	0.449
	Female	50	0.41	0.21	0.03	
**CTX1**	Male	30	3.12	2.24	0.41	0.243
	Female	50	2.56	1.74	0.25	

* Statistically significant.

**Table 4:** Correlations of the clinical parameters with the biochemical parameters.

Parameters		OHI -S	BOP	PPD	CPI	CAL
**IGF-1**	Correlation Coefficient	0.167	0.127	0.118	0.154	0.078
	Sig. (2-tailed)	0.138	0.261	0.295	0.174	0.489
**IGFBP-3**	Correlation Coefficient	0.157	0.078	0.106	0.138	−0.148
	Sig. (2-tailed)	0.164	0.493	0.350	0.224	0.189
**IGF-1/IGFBP-3**	Correlation Coefficient	0.147	0.119	0.080	0.157	0.116
	Sig. (2-tailed)	0.193	0.293	0.479	0.163	0.306
**CTX1**	Correlation Coefficient	−0.154	−0.084	−0.141	−0.173	0.190
	Sig. (2-tailed)	0.171	0.457	0.211	0.125	0.092

**Table 5:** Multiple regression of age, gender, and periodontal conditions with the levels of the biochemical parameters.

Dependent Variable	Independent Variable	Standardized Coefficients Beta	95% Confidence Interval		Sig.
			Lower Bound	Upper Bound	
**IGF-1**	(Constant)	1.353	0.584	2.123	
	Age	0.332	0.012	0.069	0.006 *
	Gender	−0.181	−0.661	0.051	0.092
	Periodontitis	−0.004	−0.259	0.251	0.974
**IGFBP-3**	(Constant)	4.185			
	Age	0.173	−0.005	0.036	0.137
	Gender	−0.375	−0.714	−0.202	<0.001 *
	Periodontitis	−0.147	−0.300	0.066	0.208
**IGF-1/IGFBP-3**	(Constant)	0.276			
	Age	0.343	0.003	0.018	0.005 *
	Gender	−0.060	−0.119	0.067	0.578
	Periodontitis	0.016	−0.062	0.071	0.895
**CTX1**	(Constant)	0.943			
	Age	0.530	0.092	0.217	<0.001 *
	Gender	−0.110	−1.223	0.342	0.266
	Periodontitis	−0.066	−0.733	0.389	0.543

* Statistically significant.

## Data Availability

Reporting data can be provided upon request.

## References

[1] Vinod K., Reddy Y.G., Reddy V.P., Nandan H., Sharma M. (2012). Orthodontic-periodontics interdisciplinary approach. J. Indian Soc. Periodontol..

[2] Khurshid Z., Zohaib S., Najeeb S., Zafar M.S., Slowey P.D., Almas K. (2016). Human saliva collection devices for proteomics: An update. Int. J. Mol. Sci..

[3] Khan R.S., Khurshid Z., Yahya Ibrahim Asiri F. (2017). Advancing Point-of-Care (PoC) Testing Using Human Saliva as Liquid Biopsy. Diagnostics.

[4] Javali M.A., Betsy J., Al Thobaiti R.S.S., Alshahrani R.A., AlQahtani H.A.H. (2020). Relationship between Malocclusion and Periodontal Disease in Patients Seeking Orthodontic Treatment in Southwestern Saudi Arabia. Saudi J. Med. Med. Sci..

[5] Lee K., Valeria B., Kochman C., Lenders C.M. (2006). Self-assessment of height, weight, and sexual maturation: Validity in overweight children and adolescents. J. Adolesc. Health.

[6] Mughal A.M., Hassan N., Ahmed A. (2014). Bone age assessment methods: A critical review. Pak. J. Med. Sci..

[7] Palanisamy V., Rao A., Shenoy R., Baranya S.S. (2016). Correlation of dental age, skeletal age, and chronological age among children aged 9-14 years: A retrospective study. J. Indian Soc. Pedod. Prev. Dent..

[8] Alijani S., Farhadian N., Alafchi B., Najafi M. (2020). Relationship of Frontal Sinus Size and Maturation of Cervical Vertebrae for Assessment of Skeletal Maturity. Front. Dent..

[9] Moca A.E., Vaida L.L., Moca R.T., Țuțuianu A.V., Bochiș C.F., Bochiș S.A., Iovanovici D.C., Negruțiu B.M. (2021). Chronological Age in Different Bone Development Stages: A Retrospective Comparative Study. Children.

[10] Ferrillo M., Curci C., Roccuzzo A., Migliario M., Invernizzi M., de Sire A. (2021). Reliability of cervical vertebral maturation compared to hand-wrist for skeletal maturation assessment in growing subjects: A systematic review. J. Back Musculoskelet. Rehabil..

[11] Cericato G.O., Bittencourt M.A., Paranhos L.R. (2015). Validity of the assessment method of skeletal maturation by cervical vertebrae: A systematic review and meta-analysis. Dentomaxillofac. Radiol..

[12] Cole T.J., Ahmed M.L., Preece M.A., Hindmarsh P., Dunger D.B. (2015). The relationship between Insulin-like Growth Factor 1, sex steroids and timing of the pubertal growth spurt. Clin. Endocrinol..

[13] Almalki A., Thomas J.T., Khan A.R.A., Almulhim B., Alassaf A., Alghamdi S.A., Joseph B., Alqerban A., Alotaibi S. (2022). Correlation between Salivary Levels of IGF-1, IGFBP-3, IGF-1/IGFBP3 Ratio with Skeletal Maturity Using Hand-Wrist Radiographs. Int. J. Environ. Res..

[14] Kanbur N.O., Derman O., Kinik E. (2005). The relationships between pubertal development, IGF-1 axis, and bone formation in healthy adolescents. J. Bone Miner. Metab..

[15] Harb A.N., Holtfreter B., Friedrich N., Wallaschofski H., Nauck M., Albers M., Meisel P., Biffar R., Kocher T. (2012). Association between the insulin-like growth factor axis in serum and periodontitis in the Study of Health in Pomerania: An exploratory study. J. Clin. Periodontol..

[16] Mishra D., Gopalakrishnan S., Arun K.V., Kumar T.S., Devanathan S., Misra S.R. (2015). Evaluation of Salivary Levels of Pyridinoline Cross Linked Carboxyterminal Telopeptide of Type I Collagen (ICTP) in Periodontal Health and Disease. Clin. Diagn. Res..

[17] Shetty S., Kapoor N., Bondu J.D., Thomas N., Paul T.V. (2016). Bone turnover markers: Emerging tool in the management of osteoporosis. Indian J. Endocrinol. Metab..

[18] Betsy J., Ahmed J.M., Mohasin A.K., Mohammed A., Nabeeh A.A.Q. (2019). Diagnostic accuracy of salivary biomarkers of bone turnover in identifying patients with periodontitis in a Saudi Arabian population. J. Dent. Sci..

[19] Choi Y.J., Lee Y.J., Lee N.Y., Lee S.H., Kim S.K., Ahn M.B., Kim S.H., Cho W.K., Cho K.S., Jung M.H. (2020). Discriminatory performance of insulin-like growth factor 1 and insulin-like growth factor binding protein-3 by correlating values to chronological age, bone age, and pubertal status for diagnosis of isolated growth hormone deficiency. Ann. Pediatr. Endocrinol. Metab..

[20] Greene J.C., Vermillion J.R. (1964). The simplified oral hygiene index. J. Am. Dent. Assoc..

[21] Trombelli L., Farina R., Silva C.O., Tatakis D.N. (2017). Plaque-induced gingivitis: Case definition and diagnostic considerations. J. Periodontol..

[22] Barbosa V.L., Angst P.D.M., Stadler A.F., Oppermann R.V., Gomes S.C. (2016). Clinical attachment loss: Estimation by direct and indirect methods. Int. Dent. J..

[23] Lee J.M., Garon E., Wong D.T. (2009). Salivary diagnostics. Orthod. Craniofac. Res..

[24] Kohl T.O., Ascoli C.A. (2017). Immunometric Double-Antibody Sandwich Enzyme-Linked Immunosorbent Assay. Cold Spring Harb. Protoc..

[25] Baccetti T., Franchi L., McNamara J.A. (2005). The Cervical Vertebral Maturation (CVM) method for the assessment of optimal treatment timing in dentofacial orthopedics. Semin. Orthod..

[26] Juul A., Dalgaard P., Blum W.F., Bang P., Hall K., Michaelsen K.F., Müller J., Skakkebaek N.E. (1995). Serum levels of insulin-like growth factor (IGF)-binding protein-3 (IGFBP-3) in healthy infants, children, and adolescents: The relation to IGF-I, IGF-II, IGFBP-1, IGFBP-2, age, sex, body mass index, and pubertal maturation. J. Clin. Endocrinol. Metab..

[27] Lo¨fqvist C., Andersson E., Gelander L., Rosberg S., Hulthen L., Blum W.F., Wikland K.A. (2005). Reference Values for Insulin-Like Growth Factor Binding Protein-3 (IGFBP-3) and the Ratio of Insulin Like Growth Factor-I to IGFBP-3 throughout Childhood and Adolescence. J. Clin. Endocrinol. Metab..

[28] Almalki A., Thomas J.T., Salama M.H., Alghamdi S.A., Almulhim B., Alassaf A., Joseph B., Alqerban A. (2022). Comparison of Salivary IGF-1, IGFBP-3, and CTX with Periodontal Status among Patients Belonging to Various Skeletal Maturity Groups. Oral Health Prev. Dent..

[29] Baim S., Miller P.D. (2009). Perspective: Assessing the Clinical Utility of Serum CTX in Postmenopausal Osteoporosis and Its Use in Predicting Risk of Osteonecrosis of the Jaw. J. Bone Miner. Res..

[30] Takenouchi Y., Ohshima M., Yamaguchi Y., Nishida T., Senda N., Idesawa M., Otsuka K., Ito K. (2010). Insulin-like growth factor-binding protein-2 and-3 in gingival crevicular fluid. J. Periodontal Res..

